# How are the Dietary Needs of Pregnant Incarcerated Women Being Met? A Scoping Review and Thematic Analysis

**DOI:** 10.1007/s10995-023-03884-1

**Published:** 2024-02-11

**Authors:** Tanya S. Capper, Adele Baldwin, Laura Abbott, Annette Briley, Rebecca Shlafer

**Affiliations:** 1 School of Nursing, Midwifery and Social Sciences, CQUniversity Australia, Level 20, 160 Ann Street, Brisbane, QLD 4000 Australia; 2 School of Nursing, Midwifery and Social Sciences, CQUniversity Australia, 538 Flinders Street, Townsville, QLD 4810 Australia; 3 Department of Allied Health and Midwifery, University of Hertfordshire, College Lane Campus, Room F305, The Wright Building, Hatfield, Hertfordshire AL10 9AB UK; 4 College of Nursing & Health Sciences, Sturt Road, Bedford Park, SA 5042 Australia; 5 Division of General Pediatrics and Adolescent Health, Department of Pediatrics, University of Minnesota, 717 Deleware St. SE, Minneapolis, MN 55414 USA

**Keywords:** Incarceration, Pregnancy, Diet, Prison, Scoping review

## Abstract

**Introduction:**

The number of incarcerated pregnant women is increasing globally. With many having complex health and social backgrounds, incarceration provides opportunities for health interventions, including the chance to have their nutritional needs met. Despite the additional nutritional requirements of pregnancy being well documented, how these are being met within the correctional setting is currently poorly understood.

**Methods:**

A scoping review of the literature was conducted to identify the literature published between January 2010 and April 2023 related to the provision of nutrition for pregnant women in the international prison systems. Sixteen papers met the criteria for inclusion in the review. The relevant key findings were charted and thematically analysed.

**Results:**

Two themes were identified: ‘the inconsistent reality of food provision’ and ‘choice, autonomy and food’. There is a clear disparity in the way in which diet is prioritised and provided to pregnant incarcerated women across several countries.

**Discussion:**

The findings highlight the need for a consistent approach to diet on a macro, global level to ensure the health of women and their infants in context.

## Significance

**What is Already Known on This Subject?:**

Whilst the additional nutritional requirements of pregnant women are well known, how these translate into the correctional setting is unknown.

**What this Study Adds?:**

This scoping review provides a synthesis of the literature related to the provision of diet for incarcerated pregnant women. The findings highlight that prisons housing pregnant and breastfeeding women should ensure that evidence-based policies exist and are enforced to ensure an appropriate diet is provided. Furthermore, flexible access to water and additional snacks between meals should be ensured to meet the women’s evolving nutritional needs across the childbearing continuum.

## Introduction

Optimising maternal and child health is a global health priority, however, inequalities in health outcomes remain for some vulnerable populations (Rao et al., 2020). Related interventions should recognise those experiencing special circumstances, such as incarcerated pregnant women, to ensure that their basic human needs are being met. Having equitable access to safe housing, social connections, relationships, and food and water are fundamental human rights which are applicable to everyone, including those who are incarcerated (Goshin et al., 2017). The United Nations Convention on the Rights of the Child (CRC) (United Nations, 1989) asserts protection of a child’s right to grow free of discrimination and confers special protection to both the mother and child for access to adequate nutrition, housing, and healthcare in order to optimise health outcomes (United Nations, 1989). This is becoming increasingly important as the number of incarcerated women continues to rise worldwide (Walmsley, 2017), and many are of young and childbearing age (Penal Reform International, 2021). Whilst incarceration is deemed punishment for crimes committed, it does however provide a unique opportunity for healthcare, support, and education to be provided to an often structurally vulnerable population who may otherwise become disengaged with healthcare services. Additionally, as pregnancy is known to be a time when women are receptive to making positive adjustments to their habits and behaviour (Bagherzadeh et al., 2021), providing appropriate care and support to this group of women has potential to improve maternal and infant outcomes in the long term.

At present, whilst the additional nutritional requirements of pregnancy are well documented, little is known about how these are being met within the correctional setting. The findings of this scoping review will provide a synthesis of the current literature in order to provide a better understanding of how pregnant women’s additional dietary needs are being met within the prison setting.

## Methods

Arksey and O’Malley’s five-stage framework (2005) underpinned this scoping review. First, the research question was identified. Second, the relevant studies were located. Third, the appropriate studies were selected and included. Fourth, the data was charted, and finally, the results were collated, summarised, and reported.

Adopting this systematic approach supported rigour and transparency allowing the relevant evidence to be systematically identified, analysed, synthesised, and presented. This enabled the breadth, depth, and nature of the existing knowledge on the provision of nutrition for incarcerated pregnant women to be comprehensively understood, and any gaps in the literature identified (Munn et al., 2018).

The 22-item checklist for the Preferred Reporting Items for Systematic Reviews and Meta-Analyses extension for Scoping Reviews (PRISMA-ScR) (Tricco et al., 2018) guided the reporting of the review.

### Identification of the Review Question

The research question is: How are the dietary needs of pregnant women in prison being met?

#### Identify the Relevant Studies

A preliminary literature search was undertaken to identify the terminology and phrases that are frequently used in this field. These were discussed, agreed upon, and used to develop the search strategy. The combination of keyword/s, phrases, Boolean operators, and truncation symbols used to form the final search strategy are presented in Table 1.

**Table 1:** Search strategy

Main keywords/phrases	Variations included
[“incarcerat*”] **AND** [“pregnan*”]	**AND** [“diet* need*” **OR** “diet* requirement*” **OR** “diet*” **OR** “nutrition*”]
[“Prison*”] **AND** [“pregnan*”]	**AND** [“diet* need*” **OR** “diet* requirement*” **OR** “diet*” **OR** “nutrition*”]
[“Jail*”] **AND** [“pregnan*”]	**AND** [“diet* need*” **OR** “diet* requirement*” **OR** “diet*” **OR** “nutrition*”]

A three-stage search process was undertaken. First, the search term combinations were systematically applied to search the full texts of the articles located within the following five predetermined databases: Web of Science, EMBASE, PubMed, Medline, and CINAHL. The databases selected are deemed the most appropriate to provide thorough coverage of the literature when undertaking a literature review (Bramer et al., 2017). Next, a follow-up search of Google Scholar was undertaken using identical search terms to identify the grey literature that had not been captured through the previous searches. Finally, the reference lists of the included articles were hand-searched for any key omissions.

Appendix 1 presents an example of the database search undertaken in CINAHL.

### Study Selection

Table 2 presents a summary of the inclusion and exclusion criteria applied to each paper captured as part of the search process.

**Table 2:** Inclusion and exclusion criteria

Included	Excluded
Primary research	Book chapters
Full-text availability	Educational resources/evaluations of educational programs
Published in English	
Published 1st Jan 2010–1st April 2023	Papers reporting on previously included data (unless otherwise specified)
All types of literature reviews	
Papers that arrived at findings or themes related to the phenomenon of interest	Papers reporting on women with incarcerated partners
Formal reports and consultation papers	Papers not focussing on pregnancy or diet
Theses	Opinion pieces and commentaries

**Fig. 1:**
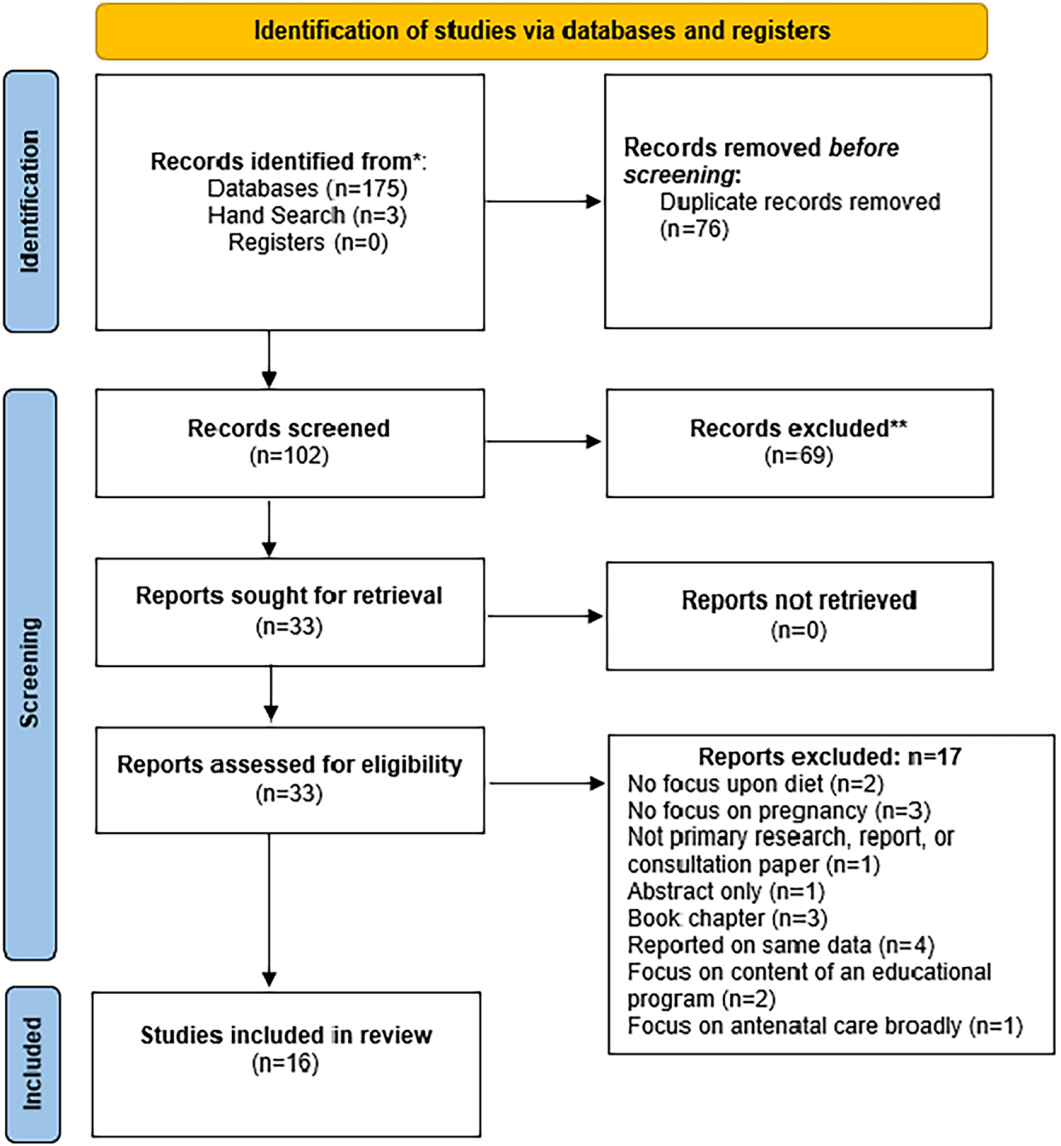
PRISMA flow chart. Adapted from Page MJ, McKenzie JE, Bossuyt PM, Boutron I, Hoffmann TC, Mulrow CD, et al (2021). The PRISMA 2020 statement: an updated guideline for reporting systematic reviews. BMJ 372:271. 10.1136/bmj.n71

To ensure all contemporary literature was captured, the search dates were limited to between January 2010 and April 2023. A total of 175 papers were identified through the database search and a further three papers were identified through the reference search. After the removal of duplicates (76), the titles and abstracts of 102 papers were screened. A total of 69 papers were then excluded and the full text of 33 were retrieved for review. All 33 papers were independently reviewed by the team and any conflicts agreed upon by consensus. Once this process was complete, sixteen papers met the inclusion criteria and formed the basis of the analysis. As quality assessment of the sources of evidence is not a required element of scoping reviews (Pollock et al., 2022), this was not completed. The PRISMA diagram (Fig. 1) presents the process by which the papers were excluded and the reasons for exclusion following full-text assessment.

### Charting the Data

The relevant data, guided by the review question; *how are the dietary needs of pregnant women in prison being met?* were extracted from each paper and charted using a standardised tool which had been previously developed by the review team. The key extracted data is presented in Table 3.

**Table 3:** Data extraction

Author/s Year of publication Country of origin	Aim/s	Population sample size	Methodology methods	Key relevant findings
Abbott (2018) UK	To examine the pregnant woman’s encounter with the English prison estate and the associated conditions	Twenty-eight (28) female prisoners that were pregnant or had recently given birth and ten staff members	Qualitative ethnography Doctoral Thesis	The quality and timing of meals were problematic. Many are hungry & lost weight The food was unappealing many carbs. Little fresh fruit and veg. Food not cooked. Snacks are supposed to be provided but aren’t. Pregnancy packs were provided in some locations but stopped postnatally. The food was squashed and soggy due to the packaging. Dehydration was reported as the water tasted unpleasant—warm and from crusty taps. Lack of money limited the opportunity to buy up extra food, but the options were often unhealthy. Eat fast in unpleasant surroundings
Abbott et al. (2023) UK	To examine pregnant women prisoners' and custody staffs' experiences and perceptions of midwifery care in English prisons	Twenty-eight (28) female prisoners. Ten (10) staff members including six (6) prison staff and four (4) healthcare personnel	Qualitative ethnography Semi-structured interviews and observational fieldwork	The healthcare personnel introduced a multivitamin tablet for all pregnant women in prison as the provided diet was perceived to be inadequate The healthcare personnel felt satisfied that whilst the women were in their care they were, at the very least, getting a multivitamin supplement
Albertson et al. (2012) UK	The aim of the consultation was to scope and map the health needs and health care of childbearing women in prison in the Yorkshire and Humberside region	Scoping review A focus group with 16 participants An expert panel of 40 participants Interviews with 5 managers	Consultation paper	Women receive inadequate food and nutrition during pregnancy. Prison staff lack nutritional expertise. The women should be provided with nutritional education Food and nutrition vary across the prison estate, and each is responsible for the nutritional needs of pregnant women. Food costing is allocated per prisoner—not increased during pregnancy. Meals are at a set time with no snacks in between. Some prisons do provide additional milk to pregnant women. One prison provided a ‘pregnancy pack’ and a dietary leaflet. A consistent approach to nutrition across prisons is required: meal plan, and clarity about entitlement to ‘Healthy start” (vitamins and milk service)
Alirezaei and Roudsari (2020) Iran	To review the guidelines that include the provision of healthcare for incarcerated pregnant women	Thirteen (13) guidelines were included	A narrative review of guidelines	Eight guidelines referred to the diet and nutrition of pregnant prisoners. Six were from the USA, one from the UK and one international (WHO). One of the guideline priorities was the nutrition and diet of pregnant women. Imprisoned women do not have control over when or what they eat and drink. They have no knowledge of the contents of their foods. Access to fluids is often inadequate. Timing and flexibility of food intake must take place. Pregnant women should be given healthy foods and snacks to address hunger in between meals
Alirezaei and Roudsari (2022a) Iran	To examine the needs of incarcerated pregnant women	Thirty-one (31) studies were included in the review	Systematic literature review	Pregnant women need adequate nutrition, activity, and rest. Nutrition is considered an essential part of antenatal care
Alirezaei and Roudsari (2022b) Iran	To understand the meaning of prenatal care in lived experience of imprisoned pregnant women	Eleven (11) pregnant and early postpartum women in prison	Qualitative. Interpretive Descriptive Phenomenology Interviews	Pregnant incarcerated women are unable to work which negatively impacts their finances and therefore their health. Women use the money earned to purchase additional food are unable to do this during pregnancy. Women rely on family to bring in extra food or provide money—those that lack this support often go hungry Food was a priority of pregnant inmates and their lack of control around this was challenging for them. The food provided was considered poor quality and inadequate and the women often found themselves asking other prisoners for food. The low nutritional value of the food concerned the women and led to concerns about the health of the unborn infant. Snacks were sometimes provided but this was inconsistent. Pregnancy cravings were not satisfied
Baldwin et al. (2020a) Australia	1. Identify pregnant women’s needs during the antenatal, birthing, and postnatal periods in prison 2. Examine how pregnant incarcerated women’s needs are met by correctional institutions 3. Explore what maternity services are available and how these services are provided	Thirty-two (32) studies were included in the review	Integrative literature review	Poor nutritional status was common across the pregnant prison population Being incarcerated led to better nutrition particularly when the sentence is longer Poor nutritional status increases the risk of miscarriage, & pregnancy and birth complications The provision of a nutritional diet varies across prisons
Bard, et al. (2016) UK	To identify effective models of care for incarcerated pregnant women	Eighteen (18) studies were included in the review	Systematic literature review	Two studies discussed the provision of additional foods (special diet) to meet the women’s nutritional requirements and the provision of vitamin and iron supplementation. Some prisons did not provide any nutritional services. One prison provided additional foods to meet nutritional needs during pregnancy and provided additional snacks. ‘Health education’ classes available. Dietary intake is a modifiable risk factor that can be targeted/addressed whilst in prison
Ferszt and Clarke (2012) USA	To examine the health care practices of pregnant women in state prisons	Wardens from 19 different facilities	Mixed methods. Survey: 62 multiple choice and four open ended questions	In many state prisons, nutritional recommendations for a healthy pregnancy are not met. Extra foods provided daily: Milk, Cereal, Fruit, Peanut Butter, and Evening snack. The nutritional needs of pregnant women are only partially met by the majority of prisons, many of which reported a limited diet of fruits and vegetables. It is recommended that women meet with a nutritionist to discuss and plan a healthy diet to meet the nutritional standards for pregnant women A written nutritional educational pamphlet for pregnant women is an An additional resource can be used. One prison has a doctor that prescribes a multivitamin and a nutritional consult
Hackett (2017) USA	To provide insight into the nutritional, physical activity, and stress management experiences of incarcerated pregnant women to tailor/adjust a health program to better meet their needs	Ten (10) women, (6 of whom were pregnant whilst incarcerated). Three (3) birthed whilst incarcerated	Qualitative Face-to-face interviews Master’s Thesis	There is a need for more nutritious food options within the prison. Clients were very dissatisfied with their meals: Food wasn’t cooked properly—which caused illness. Meals are small, cold, poor tasting, nutritionally insufficient and repetitious. Unhealthy and inadequate. Given a daily ‘pregnancy bag’ but needed to substitute with food from ‘the commissionary” food—unhealthy. Night snack and a pregnancy snack. Lacked healthy options. No fruit etc. Gained weight
Ifeonu et al. (2022) Canada	To contemplate and demarcate food’s different valuations in prison	Five hundred (500) Incarcerated individuals	Qualitative Interviews	Having three meals a day in prison is a bonus when pregnancy. Women didn’t often eat when on the ‘outside’ and felt unsafe. Regular meals and access to food is a benefit of incarceration. Not having to worry where the next meal is coming from is positive when pregnant. Women give the pregnant women extra food and milk to take care of them
Kelsey et al. (2017) USA	To examine the pregnancy-related accommodations and health care provided for regional jail populations	Employees of 53 jail medical facilities across the US	Quantitative. Survey	Fluid, vitamins, and food supplements were provided for pregnant women in 50–60% of cases. 51% of prisons provided healthier food options. A packaged meal is provided. The lack of healthy food options is an example of a lack of adherence to care standards for pregnant incarcerated women
Kirubarajan et al. (2022) Canada	To characterise patient experiences regarding pregnancy and childbirth during incarceration	Twenty-four (24) studies were included in the review	Qualitative systematic literature review	40% of officers do not believe that pregnant women should be provided with special accommodations whilst incarcerated (i.e., different diet). Pregnant incarcerated women were surprised that they did not receive healthy snacks. All pregnant inmates should receive dietary supplements throughout pregnancy and breastfeeding
Kotlar et al. (2015) USA	N/A	N/A	Report Challenges and opportunities	Many prisons lack nutritional policies and often standards for nutrition are not followed. Nutrition information is shared with women by ‘Motherhood Behind Bars’. Policies are necessary to ensure the health of pregnant women—this includes nutrition
Kramer et al. (2023) USA	To assess prison and jail pregnancy policies and practices with an emphasis on restraint use and compliance with anti-shackling legislation	Twenty-two (22) state prisons and 6 jails	Survey of policies related to correctional practices for pregnant prisoners	All facilities provide vitamin supplements and most additional food and snacks that are high in protein. Some facilities had policies on pregnant women’s additional calorific needs (pregnancy specific diet) and the types of foods that are to be provided. These included yoghurt, cheese, vegetables, sandwiches, and peanut butter Some facilities provide additional milk and fresh fruit for pregnant women
Nair et al. (2021) USA	To examine prenatal care provided to incarcerated women identifying areas where improvement is required. To examine current legislative gaps to ensure uniform templates of care are provided at women’s prisons	Not described	Literature review	No federal regulations set a national standard for the nutrition of pregnant inmates. Many prisons have no minimum standards regarding pregnancy nutrition or supplementation for incarcerated women. Pregnant prisoners should be provided with a diet supplemented with the necessary vitamins and minerals. This should be mandated. Excessive nausea and vomiting must be appropriately managed in order to ensure adequate maternal nutrition is maintained. Healthy foods are expensive and therefore the meals provided to pregnant inmates may not be adequate. Pregnant women should drink up to three litres of fluid a day

### Collating, Summarizing, and Reporting the Results

The data was collated in a Microsoft Excel spreadsheet allowing the summary of each paper to be compared and contrasted in context. It is to be acknowledged that whilst scoping reviews typically lend themselves to data analysis methods that allow the descriptive presentation of results (Arksey and O’Malley 2005), due to the nature of the topic of interest and the review question, thematic analysis, guided by Saldaña’s (2016) standalone analysis method *Theming the Data* was selected. This method allowed the coding, identification, and allocation of thematic phrases to the groups of data, in order to report the findings (Saldaña, 2016). Selecting a thematic rather than content analysis approach enables data to be contextually synthesised and presented, conveying a deeper understanding of the literature on this topic (Onwuegbuzie et al., 2016). The themes identified were discussed amongst the team, refined, and agreed upon by consensus.

## Results

Of the sixteen included papers, six were from the United States of America (USA) (Ferszt & Clarke, 2012; Hackett, 2017; Kelsey et al., 2017; Kotlar et al., 2015; Kramer et al., 2023; Nair et al., 2021), four from the United Kingdom (UK) (Abbott, 2018; Abbott et al., 2023; Albertson et al., 2012; Bard et al., 2016), three from Iran (Alirezaei & Roudsari, 2020, 2022a, 2022b), two from Canada (Ifeonu et al., 2022; Kirubarajan et al., 2022), and one from Australia (Baldwin et al., 2020a). Five of the included papers were literature reviews (Alirezaei & Roudsari, 2022a; Baldwin et al., 2020a; Bard et al., 2016; Kirubarajan et al., 2022; Nair et al., 2021), one a mixed methods study (Ferszt & Clarke, 2012), one a quantitative study (Kelsey et al., 2017), five qualitative studies (Abbott, 2018; Abbott et al., 2023; Alirezaei & Roudsari, 2022b; Hackett, 2017; Ifeonu et al., 2022), two policy analyses (Alirezaei & Roudsari, 2020; Kramer et al., 2023), one a multi-component consultation paper (Albertson et al., 2012), and one a report (Kotlar et al., 2015).

### Themes

Two main themes were identified: *‘the inconsistent reality of food provision’* and *‘choice, autonomy and food.* Each will now be discussed in greater detail.

### Theme 1: The Inconsistent Reality of Food Provision

The theme, *the inconsistent reality* of food provision highlights the clear disconnect between rhetoric and reality of operationalising organisational policies relating to food provision for pregnant women in prison. The majority of the sixteen papers acknowledged the additional nutritional requirements of pregnant women which were broadly based upon local government dietary guidelines. Despite the provision of adequate nutrition being recognised as an essential component of antenatal care, the way in which these recommendations were translated into correctional practice was inconsistent.

Several papers identified the need for consistent dietary policies and guidelines that are specific to pregnant women within the correctional setting (Alirezaei & Roudsari, 2020; Kelsey et al., 2017; Kotlar et al., 2015; Nair et al., 2021). Whilst some prisons have developed such policies, often they were inconsistently followed (Albertson et al., 2012; Ferszt & Clarke, 2012; Kelsey et al., 2017; Kotlar et al., 2015) and in some settings, no dietary guidance was in place for this key population of women (Albertson et al., 2012; Alirezaei & Roudsari, 2020, 2022a, 2022b; Kotlar et al., 2015; Nair et al., 2021). The absence of, or poor adherence to dietary policies appears to be one of the key contributing factors toward the inconsistent provision of a nutritional diet for pregnant women within the prison services captured in this review.

Several additional factors also appear to influence prison services’ ability to provide healthy and nutritious food for incarcerated pregnant women. The broader literature suggests that good nutritional intake is often not seen as a prison priority (Reese & Sbicca, 2022), particularly when facing increasing financial challenges due to budget cuts and the rising costs of food. Despite this, the need to feed a large population remains and has led to an allocated ‘per head’ food budget which often does not increase for pregnant women (Albertson et al., 2012). The cost of ingredients, therefore, plays an important role in determining both the type and quantity of food offerings for the greater prison population, including pregnant women (Nair et al., 2021).

Despite one paper highlighting that 40% of prison officers do not believe that pregnant women should be treated any differently to the other inmates, inclusive of the food they were provided with (Kirubarajan et al., 2022), five studies reported that pregnant women were routinely provided with additional food or snacks (Alirezaei & Roudsari, 2020; Bard et al., 2016; Ferszt & Clarke, 2012; Hackett, 2017; Kramer et al., 2023), and others were given a ‘pregnancy pack’, bag or vouchers (Albertson et al., 2012; Hackett, 2017; Kelsey et al., 2017). The specific contents of the ‘pregnancy pack’ differs between institutions and are not normally itemised. Generally, these packs include additional sources of nutrition such as extra food (e.g., bread) and/or milk or milk supplements. The two studies by Abbott (2018) and Alirezaei & Roudsari (2022b) discussed how women could ‘buy up’ extra foods, but lack of funds often limited this (Abbott, 2018), particularly when they were not allowed to work (Alirezaei & Roudsari, 2022b), and the foods available to buy were often unhealthy (Abbott, 2018). A complete lack of extra food offerings was lamented in other studies (Albertson et al., 2012; Alirezaei & Roudsari, 2022b; Kirubarajan et al., 2022).

The food that was provided was reported to be of poor quality, stodgy and either under or overcooked in three studies (Abbott, 2018; Alirezaei & Roudsari, 2022b; Hackett, 2017) and several papers reported on the importance of providing vitamin supplements (Abbott et al., 2023; Albertson et al., 2012; Ferszt & Clarke, 2012; Kelsey et al., 2017; Kirubarajan et al., 2022; Kramer et al., 2023; Nair et al., 2021). These were seen as an easy way to ensure pregnant women’s nutritional needs were met. Consideration of individual dietary needs, whether for medical, religious, or individual reasons, was a challenge (Nair et al., 2021). Furthermore, the needs of women experiencing symptoms associated with pregnancy such as nausea and vomiting, pica, and gastroesophageal reflux were not considered or accommodated (Alirezaei & Roudsari, 2022b; Nair et al., 2021). Additionally, in some cases, it was indirectly implied that eating healthily was in part the responsibility of the women; they were provided with advice and leaflets on healthy eating during pregnancy (Albertson et al., 2012; Ferszt & Clarke, 2012), whilst others received educational classes on how to be healthy during pregnancy (Albertson et al., 2012; Bard et al., 2016; Kotlar et al., 2015). As prison meals are generally prepared in commercial-style kitchens designed to produce meals for large numbers of people, there was little scope for the consideration of individual dietary needs or choices, a consistent finding across the reviewed literature.

Adequate access to fluids was widely recognised as being important during pregnancy. Women were believed to require several litres of water each day to maintain healthy levels of hydration (Nair et al., 2021), however, access to a water source was sometimes restricted (Alirezaei & Roudsari, 2020). In other cases, the water was unappealing to the women due to its taste, temperature, and the crusty taps they were expected to drink from (Abbott, 2018). Just three papers referred to the provision of additional milk for pregnant women (Albertson et al., 2012; Ferszt & Clarke, 2012; Kramer et al., 2023).

Despite the importance of a healthy dietary intake during pregnancy being widely recognised within the correctional context, incarcerated pregnant women were generally perceived to have a poor nutritional status, which improved when the custodial sentence was longer (Baldwin et al., 2020a). This suggests that in spite of the dietary deficits identified in the literature, being incarcerated does in fact often *improve* the pregnant women’s nutritional intake from that consumed prior to sentencing (Ifeonu et al., 2022). So, whilst the literature identified a range of shortfalls and inconsistencies related to the dietary intake of pregnant incarcerated women, it was also discussed within the social context, reflecting upon the social role food plays including the social process of eating/dining.

### Theme 2: Choice, Autonomy, and Food

The majority of the included papers either directly or indirectly referred to the way in which being incarcerated removed the women’s autonomy around food choices, food quality, portion sizes, access to snacks and water, and when and where they eat. These restrictions appeared to influence the appeal and the inherent social aspects of eating and drinking. The second theme *choice, autonomy and food* reflects the social nature of food and how the processes associated with providing food and the eating environment shape how, whether and what an individual eats.

As those from disadvantaged backgrounds make up the majority of women in prison (Baldwin et al., 2020a), many lack food literacy and are therefore more likely to have a distorted understanding of what constitutes an appropriate diet during pregnancy or otherwise. That is, fresh fruit, vegetables and healthy protein sources may not be a pre-existing part of the food repertoire for these women. With this in mind, the women’s dietary intake was perceived to be far better whilst incarcerated in terms of the nutritional value of the foods they consumed and the amount and frequency of their meals compared to when ‘outside’ (Baldwin et al., 2020a; Ifeonu et al., 2022). However, despite prison providing an opportunity for women to learn about healthy eating and have their additional nutritional needs met, their lack of autonomy often meant that they were unable to access the information required to make decisions about the foods they were consuming. For example, the women were unable to read the food packaging to determine its composition or nutritional value (Alirezaei & Roudsari, 2020), and with many having little or no existing nutritional knowledge other than that provided in some prisons, identifying healthy options was a challenge.

The food provided was described as unappealing, inadequate, squashed, and soggy due to its packaging and an abundance of carbohydrate-rich foods was provided with few fruit and vegetable options (Abbott, 2018; Alirezaei & Roudsari, 2022b; Ferszt & Clarke, 2012; Hackett, 2017; Kelsey et al., 2017; Nair et al., 2021). This led to women feeling that they were simply ‘being fed’ rather than nourished when they ate (Abbott, 2018). These unappealing, repetitive food offerings coupled with the regimented timing of meals left some women feeling hungry and they lost weight (Abbott, 2018). Additionally, the meals were cold at the time of serving, and women worried about whether they may become ill (Abbott, 2018; Hackett, 2017).

There is little opportunity for incarcerated people to control what they do and when they do it; that is the nature of incarceration and pregnant women are treated no differently. This extends to the ability to access, prepare, and consume appealing healthy foods and drinks outside of prescribed mealtimes in the institutions (Albertson et al., 2012; Alirezaei & Roudsari, 2020). The women also lacked the autonomy to decide where they ate, and some were forced to eat close to cell toilets and around other prisoners meaning that they often felt compelled to eat fast (Abbott, 2018). These factors had distinct influences on the women’s decisions about what, when and how to eat and also whether they enjoyed their meals and felt that their hunger was satisfied across each 24-h period. This then in turn influenced how the women’s nutritional needs were being met.

## Discussion

Prisons are rarely designed for women and are often seen as a ‘plus one’ to penal systems fundamentally designed for men (Boppre, 2019). Recognition of the specific needs of incarcerated women needs to go beyond the psychosocial or illness models; considering the unique needs of pregnant women in prison must guide policy and practice to improve outcomes. Prisons are regimented and complex environments where women experience limited support during pregnancy, birth, and during their child’s early life and this includes access to quality nutrition. Current disconnections between recommendations and reality negatively influence maternal wellbeing (Breuer et al., 2021) and may increase the risk of miscarriage, and pregnancy and birth complications.

Collaborative approaches with charities such as Birth Companions have resulted in the publication of birth charters, one for women in prison in England and Wales (Kennedy et al., 2016) and another for women in prison in Australia (Baldwin et al., 2020b). Corrective services across this footprint were involved in the development of these charters and have considered their implementation. Of note, is the lack of detailed discussion about nutrition for pregnant women in prison, which, in hindsight for the authors, may guide future iterations.

Globally, incarcerated women are already over-represented in the poor outcomes associated with the social determinants of health (Rao et al., 2020), with multiple, complex, intergenerational impacts. Poor nutrition not only affects the health of the foetus and infant, but may contribute to lifelong, chronic conditions, physical and psychological limitations. Across countries, communities, and backgrounds, many women in prison come from dysfunctional homes with a backdrop of anti-social behaviour and violence, low levels of educational attainment, and poor health across their lifespan (Baldwin et al., 2022). Incarceration, therefore, provides an opportunity to promote a positive pregnancy and parenting experience and should include consideration of all the factors that promote health and wellbeing—including ensuring a healthy diet. However, if not managed appropriately, there is a risk of exacerbating vulnerabilities, and contributing to poorer health and wellbeing across generations. Unfortunately, these potential impacts would be felt around the world, creating a global at-risk population group.

Whilst it is acknowledged that at present the scope to improve the food quality may be limited due to global financial constraints, however, upwards adjustments must be made to the funding allocation for meals for pregnant women, rather than taking a one size fits all approach to feeding the prison population.

## Limitations

Six of the sixteen papers captured were literature reviews that had incidentally arrived at findings related to the diet of pregnant incarcerated women. Therefore, at present, only a small amount of literature exists specifically exploring this topic. This lack of research may be due to the ethical challenges associated with conducting research with incarcerated women. The geographical distribution of the authors of the included papers were limited to the UK, Iran, Australia, and North American countries. This may limit the transferability of the findings to other contexts. Further research is needed to address these limitations and provide more comprehensive insights into this critical issue.

## Conclusions for Practice

Multiple complex external factors frame how prisons provide nutrition for pregnant women. A fundamental element of antenatal care in this context is to ensure that both women and custodial staff are provided with education to improve their understanding of nutritional needs during pregnancy and beyond. The challenge with this, however, is the incarcerated woman’s inability to access appropriate food to ensure her physical needs during pregnancy are being met. In some correctional settings policies and frameworks do exist to guide correctional practices regarding the dietary intake of pregnant women, but the way in which these are implemented is dependent upon individual organisational practices. Future clinical practice and organisational policies must address the existing disparity in the provision of evidence-based care during pregnancy which includes equitable access to appropriate nutrition for all pregnant women in prison around the world in context, regardless of location.

## Conclusion

In most cases, pregnant women in prison have complex health and social needs and therefore the importance of good nutrition cannot be underestimated. Ensuring that incarcerated pregnant women are provided with nutritious meals is however a multifaceted challenge, influenced by various external factors. Incarceration provides opportunities for health interventions, education, and support, and needs to form part of antenatal care, incorporating women and custodial staff. Further research into the nutritional wellbeing of all women in prison is required, but a cornerstone on pregnant women is vital for the overall wellbeing of both the woman and her infant. Supporting a healthy start for women and their infants encourages healthy attitudes towards food which, once released from prison, may improve, and sustain long term health.

## Appendix 1: Example Database Search

01 Jan 2010 to 01 Apr 2023

**Table:** 

Database	Search terms (Title and Abstract)
**CINAHL** (n = 42)	[Incarcerat*] **AND** [“pregnan*”] **AND** [“diet* need*” **OR** “dietary requirement*” **OR** “diet*” **OR** “nutrition*”] [“Prison*”] **AND** [“pregnan*”] **AND** [“diet* need*” **OR** “dietary requirement*” **OR** “diet*” **OR** “nutrition*”] [“Jail*”] **AND** [“pregnan*”] **AND** [“diet* need*” **OR** “dietary requirement*” **OR** “diet*” **OR** “nutrition*”]

## Data Availability

Not applicable.
